# Approaches and Methods to Measure Oxidative Stress in Clinical Samples: Research Applications in the Cancer Field

**DOI:** 10.1155/2019/1279250

**Published:** 2019-03-12

**Authors:** Meghri Katerji, Maria Filippova, Penelope Duerksen-Hughes

**Affiliations:** Department of Basic Sciences, Loma Linda University School of Medicine, 11021 Campus Street, 101 Alumni Hall, Loma Linda, CA 92354, USA

## Abstract

Reactive oxygen species (ROS) are common by-products of normal aerobic cellular metabolism and play important physiological roles in intracellular cell signaling and homeostasis. The human body is equipped with antioxidant systems to regulate the levels of these free radicals and maintain proper physiological function. However, a condition known as oxidative stress (OS) occurs, when ROS overwhelm the body's ability to readily detoxify them. Excessive amounts of free radicals generated under OS conditions cause oxidative damage to proteins, lipids, and nucleic acids, severely compromising cell health and contributing to disease development, including cancer. Biomarkers of OS can therefore be exploited as important tools in the assessment of disease status in humans. In the present review, we discuss different approaches used for the evaluation of OS in clinical samples. The described methods are limited in their ability to reflect on OS only partially, revealing the need of more integrative approaches examining both pro- and antioxidant reactions with higher sensitivity to physiological/pathological alternations. We also provide an overview of recent findings of OS in patients with different types of cancer. Identification of OS biomarkers in clinical samples of cancer patients and defining their roles in carcinogenesis hold great promise in promoting the development of targeted therapeutic approaches and diagnostic strategies assessing disease status. However, considerable data variability across laboratories makes it difficult to draw general conclusions on the significance of these OS biomarkers. To our knowledge, no adequate comparison has yet been performed between different biomarkers and the methodologies used to measure them, making it difficult to conduct a meta-analysis of findings from different groups. A critical evaluation and adaptation of proposed methodologies available in the literature should therefore be undertaken, to enable the investigators to choose the most suitable procedure for each chosen biomarker.

## 1. Introduction

Reactive oxygen species (ROS) are chemically reactive molecules containing oxygen that play several beneficial roles for the organism. At low/moderate concentrations, they are needed for physiological activities such as intracellular cell signaling and homeostasis, cell death, immune defense against pathogens, and induction of mitogenic response [1–5]. These free radicals are produced endogenously as a natural by-product of the normal cellular metabolism of oxygen. Additionally, they can be induced by exogenous sources such as UV light, ionizing radiation, lifestyle, diet, stress, and smoking. Maintaining equilibrium between the reducing and oxidizing states is crucial for proper physiological functions; therefore, living organisms are equipped with antioxidant defense systems, consisting of both enzymatic and nonenzymatic antioxidants, to regulate the levels of these free radicals [6–11].

An imbalance between the production of ROS and the ability of the antioxidant systems to readily detoxify these reactive intermediates results in oxidative stress. Free radicals generated in excessive and uncontrollable amounts under oxidative stress conditions cause damage to DNA, proteins, and lipids, which can severely compromise cell health and contribute to disease development [12–14]. Indeed, in the past years, considerable research has demonstrated that oxidative stress is involved in the natural process of aging as well as a wide variety of human diseases, including neurodegenerative disorders, multiple sclerosis, cardiovascular disease, rheumatoid arthritis, and cancer [15–22]. Consistent with this relationship between oxidative stress and human disease, numerous studies have suggested that an increase in dietary antioxidant intake reduces the risk for coronary heart disease [23], Alzheimer's disease [24–26], Parkinson disease [27], ischemic stroke [28–31], and asthma [32].

Biomarkers of oxidative stress are therefore important tools in the assessment both of disease status and of the health-enhancing effects of antioxidants in humans. In this review, we aim to discuss different methods and approaches used for the evaluation of oxidative stress in clinical samples, as well as to review recent findings of oxidative stress in patients with different types of cancer.

## 2. Methods and Approaches to Measure Oxidative Stress in Clinical Samples

### 2.1. Direct Measurement of Reactive Oxygen Species

Reactive oxygen species (ROS) are the key molecules responsible for the deleterious effects of oxidative stress. Direct measurement of their cellular levels is therefore one approach to determine oxidative stress conditions.

One way to estimate the cellular levels of ROS is through the use of fluorogenic probes [33–41]. Hydrogen peroxide (H_2_O_2_), hydroxyl radicals (OH^−^), and peroxyl radicals (ROO^−^) can be measured following staining with 5-(and -6)-carboxy-2′,7′-dichlorodihydrofluorescein diacetate (DCFDA). This membrane-permeable probe diffuses into the cells where it becomes hydrolyzed by intracellular esterase to DCFH. The latter remains trapped within the cells and reacts with H_2_O_2_, generating the fluorescent 2′,7′-dichlorofluorescein (DCF). Therefore, the amount of peroxide produced by the cells can be estimated by the fluorescence intensity of DCF (*λ*_excitation_ = 488 nm and *λ*_emission_ = 530 nm) as analyzed by flow cytometry or by employing a fluorescence plate reader [39]. On the other hand, superoxide molecules (O_2_^−^) can be detected following staining with another fluorescent probe, dihydroethidium (DHE). The sodium borohydride-reduced form of ethidium bromide is also permeable to viable cells. Inside the cells, DHE is directly oxidized to ethidium bromide by the superoxide anion, which then fluoresces. The red fluorescence, measured using an excitation of 488 nm and an emission of 585 nm, is therefore considered to be proportional to the intracellular superoxide anion levels [34, 38]. Another way to quantify ROS molecules such as hydroperoxides (R-OOH), particularly in serum, is by assessing the derivatives of reactive oxygen metabolites (D-Roms) test, as described by Trotti et al. [42]. In this assay, a small amount of patient serum is dissolved in an acetate-buffered solution (pH 4.8). Transition metal ions (Fe^2+^, Fe^3+^), liberated from the proteins in the acidic medium, react with hydroperoxide groups converting them into alkoxy (R-O^•^) and peroxy (R-OO^•^) radicals by way of the Fenton reaction. These newly formed radicals become trapped chemically with a chromogen (*N*,*N*-diethyl-para-phenylendiamine) leading to the formation of the corresponding radical cation. The concentrations of these newly formed radicals, which are directly proportional to those of the peroxides present in serum, are then determined by spectrophotometric procedures at an absorption of 505 nm [42–45].

### 2.2. Assessment of Oxidative Damage

Direct measurement of ROS levels with high accuracy and precision is difficult due to their short lifespan and rapid reactivity with redox state regulating components. While peroxyl radicals and hydrogen peroxide are relatively stable molecules (with half-lives of seconds to minutes), hydroxyl radicals are very reactive (having a half-life of less than a nanosecond) [46–49]. Therefore, indirect measurement of ROS by examining the oxidative damage these radicals cause to the lipids, proteins, and nucleic acids of the cells is a promising alternative approach to assess oxidative stress in clinical samples.

#### 2.2.1. Protein Damage

Protein carbonyl (PC) content is a commonly used marker of oxidative modification of proteins, providing significant evidence of oxidative stress in clinical samples. PCs are generated due to the oxidation of protein backbones and amino acid residues such as proline, arginine, lysine, and threonine by ROS molecules [50]. The oxidized proteins can be measured using the 2,4-dinitrophenylhydrazine (DNPH) method as described by Levine et al. [51], and simplified by Mesquita et al. [52]. In this assay, DNPH reacts with PCs, forming a Schiff base to produce dinitrophenylhydrazone products, the levels of which can be analyzed spectrophotometrically at 375 nm and correlated to the levels of oxidized proteins [51–54]. Alternatively, PC contents can be identified by 2D gel electrophoresis and western blot [55, 56], or by OxyBlot according to Butterfield et al. [57, 58].

The detection of advanced oxidation protein products (AOPP), according to Witko-Sarsat et al., is another approach to assess protein oxidation in clinical samples. AOPP, also defined as dityrosine containing cross-linked protein products, are generated through the reaction of plasma proteins with chlorinated oxidants such as chloramines. In this method, plasma or serum of patients, calibrated with chloramine-T, is mixed with potassium iodide and acetic acid and the absorbance is spectrophotometrically read at 340 nm [59, 60].

#### 2.2.2. Lipid Damage

Lipid peroxidation has been commonly used as an indicator of ROS-mediated damage to cell membranes. Malondialdehyde (MDA) is one of the best studied end-products of peroxidation of polyunsaturated fatty acids in clinical samples and is frequently used to estimate oxidative stress conditions [61]. The levels of MDA can be measured using thiobarbituric acid reactive substances (TBARS) [62–67] as described by Donnan [68], Yagi [69], Mihara and Uchiyama [70], Buege and Aust [71], Ohkawa et al. [72], or Yoshioka et al. [73]. In all these methods, MDA reacts with TBARS in acidic medium at 100°C to generate a pink/red-colored product which can be extracted with butanol and measured using a spectrophotometer at an absorbance of 520-535 nm or by a fluorimeter at *λ*_excitation_ = 515 nm and *λ*_emission_ = 555 nm. The TBARS method is rapid and easy; however, aldehydes other than MDA may also react with TBARS, producing derivatives that absorb light in the same wavelength range [74]. Alternatively, plasma MDA can be measured using high-performance liquid chromatography (HPLC) employing a C18 reversed-phase column [75] as described by Victorino et al. [76] or by gas chromatography-mass spectrometry (GC-MS) on a capillary column following transmethylation with sodium methoxide [77–79]. While this method determines MDA levels more reproducibly and reliably, the individual sample processing makes it time-consuming, labor-intensive, and impractical.

Other lipid peroxidation markers include 8-iso-prostaglandin F2*α* (8-iso-PGF2*α*), 4-hydroxy-2-nonenal (4-HNE), conjugated dienes (CD), and lipid hydroperoxides (LOOH), providing different reliable approaches for the identification of oxidative damage to the cell's lipids. 8-Iso-PGF2*α*, generated as a result of nonenzymatic peroxidation of arachidonic acid in membrane phospholipids, can be measured using rapid ultra-high-performance liquid chromatography-tandem mass spectrometry, noting the limitations of being labor-intensive and requiring specialized and expensive instrumentation [80, 81]. Unsaturated hydroxyalkenal 4-HNE can be investigated in tissues, preferably using immunohistochemistry (IHC), or HPLC [82, 83]. CDs, produced as a consequence of free radical-induced autoxidation of polyunsaturated fatty acids (PUFAs), can be detected according to Suryanarayana Rao and Recknagel, which follows the maximal absorption of UV light at 233 nm by these compounds [84–86]. Finally, LOOHs, which are the primary oxidation products of PUFAs, can be determined by the ferrous oxidation xylenol orange (FOX) assay, based on the ability of LOOH to oxidize ferrous iron in the presence of xylenol orange, leading to the formation of a colored ferric-xylenol orange complex, with an absorbance at 560 nm [87, 88].

Oxidized levels of low-density lipoproteins (LDL) are also sometimes measured in human serum or plasma as established biosensors of oxidative stress, using a sandwich ELISA based on the proprietary mouse monoclonal antibody 4E6, which is directed against a conformational epitope in oxidized ApoB-100. However, this method may fall short on antibody specificity for oxLDL as native LDL may also be detected [89–91].

#### 2.2.3. DNA Damage

8-Hydroxy-2′-deoxyguanosine (8-OHdG) is one of the major oxidative modifications in DNA that is generated by hydroxylation of the deoxyguanosine residues. 8-OHdG residues can be excised from the DNA by enzymatic repair systems, leading to their circulation in the blood and subsequent excretion in the urine [92]. Levels of 8-OHdG in blood and/or urine of patients can therefore be measured as a marker of oxidative DNA damage. 8-OHdG is frequently examined using HPLC coupled with an electrochemical detector (ECD) [93–96] based on the procedures elaborated by Shigenaga et al. [97], Toyokuni et al. [98], or Helbock et al. [99]. In spite of its sensitivity and accuracy, the HPLC-ECD method is not very convenient for analyzing 8-OHdG contents in clinical samples because of its cost, technical involvement, and low throughput [100]. Alternative and simpler ways to measure this DNA damage marker include the enzyme-linked immunosorbent assay (ELISA) [55, 82, 100–102] and immunohistochemical analysis [82, 101–105]. 8-oxodG can also be identified by OxyDNA-FITC conjugate binding followed by analysis by flow cytometry for fluorescence at *λ*_excitation_ = 495 nm and *λ*_emission_ = 515 nm [36].

Thymidine glycol (TG) is another principal DNA lesion caused by oxidative stress. TG is a more specific marker for oxidative DNA damage because thymidine, unlike guanosine, is not incorporated into RNA. Moreover, TG is sustained in tissues while 8-OHdG is rapidly excised from DNA and excreted in urine. Therefore, TG is an appropriate marker for oxidative DNA damage in tissue specimens. Accumulation of TG in tissues can be examined immunohistochemically using the streptavidin-biotin-peroxidase complex method [44].

Single- or double-stranded breaks within the DNA are also generated during oxidative stress conditions. These oxidative DNA lesions can be identified using the comet assay, which is based on the ability of cleaved DNA fragments to migrate out of the nucleus when an electric field is applied, unlike the undamaged DNA which migrates slower and remains within the nucleoid. Assessment of the DNA “comet” tail shape and migration pattern can therefore be used to evaluate DNA damage within cells [35, 36].

More generally, various modified DNA bases in clinical samples can also be measured using gas chromatography-mass spectrometry with selected ion monitoring (GC/MS-SIM) as described by Dizdaroglu [106]. Here, the quantification of these products is done by isotope-dilution mass spectrometry using their stable isotope-labeled analogues as internal standards.

It is worth mentioning that the DNA base modifications detected with all these methods, although very important, do not provide information as to whether the damage is in active genes or in quiescent DNA. However, it seems likely that the “exposed” and active DNA would be more sensitive to oxidative damage than that packaged into condensed chromatin.

Alternatively, DNA repair enzymes, such as human 8-oxoguanine-DNA-glycosylase (hOGG) and apurinic/apyrimidinic endonuclease (APE), which repair the endogenous DNA damage induced by increased ROS levels, can be evaluated to estimate oxidative damage in clinical samples. Their levels can be determined by IHC analysis [107, 108] or HPLC [109] while the activity of the hOGG enzyme can be assessed according to the method of Yamamoto et al. [110], which uses a double-stranded 22 bp oligonucleotide substrate containing one 8-OHdG paired with deoxycytidine at the complementary strand and labeled with FITC.

### 2.3. Assessment of Antioxidant Status

The human body is equipped with an antioxidant system that serves to counterbalance the deleterious effects of oxidative free radicals. When the balance between antioxidants and ROS species, referred to as redox homeostasis, is disturbed, oxidative stress can occur. The disturbance of this prooxidant and antioxidant balance can be a result of increased free radical production, antioxidant enzyme inactivation, or excessive antioxidant consumption. Assessment of the antioxidant status can thereby be correlated to the extent of oxidative stress in clinical samples.

Redox homeostasis is regulated by two arms of antioxidant machineries: enzymatic components and nonenzymatic, low molecular compounds. Several approaches have been developed to measure the different activities or levels of these antioxidants. Alternatively, the total antioxidant status can be evaluated to assess the oxidative state of clinical samples.

#### 2.3.1. Enzymatic Antioxidants

*(1) Superoxide Dismutase*. Superoxide dismutase (SOD) is a family of antioxidant enzymes that regulate ROS levels by catalyzing the conversion of superoxide to hydrogen peroxide and molecular oxygen [61].

Their total activity can be determined using a method described by McCord and Fridovich [111] or Misra and Fridovich [112]. The basis of this method is the ability of SOD to inhibit the autoxidation of adrenaline to adrenochrome in a basic medium, which can be measured at an absorption of *λ* = 480 nm [66, 67]. Another method to directly measure the activity of SOD has been established by Marklund and Marklund [113] and described by Roth and Gilbert [114] and is based on a similar principle. Instead of epinephrine autoxidation, their method conversely investigates the ability of SOD to inhibit the autoxidation of pyrogallol into a yellow solution that can be measured at an absorbance of 420 nm [75].

Alternatively, SOD activity can be measured using indirect methods developed by Nishikimi et al. [115] & Kakkar et al. [116] or Oyanagui (1984) & Sun et al. (1988) [117, 118]. The principle of this indirect method is that superoxide radicals, generated by the NADH/D-amino acid oxidase-phenazine methosulfate (PMS) system or the xanthine-xanthine oxidase system, respectively, cause the reduction of tetrazolium salts such as 2-(4-idophenyl) 3-(4-nitrophenol)-5-phenyltetrazolium (INT), 3′-{1-[(phenylamino)-carbonyl]-3,4-tetrazolium}-bis (4-methoxy-6-nitro)benzenesulfonic acid (XTT), or nitro blue tetrazolium (NBT), into blue formazan which can be measured spectrophotometrically at 470-560 nm. The SOD in the samples competes for the generated superoxide radicals, thereby inhibiting the reaction of tetrazolium reduction [77–79, 119–123]. In all the methods discussed above, a unit of SOD is generally defined as the amount of the enzyme which causes inhibition of the reaction (autoxidation of adrenaline, autoxidation of pyrogallol, or tetrazolium reduction).

It is worth noting that these methods can also be applied following a separation step in sample preparation to determine the different activities of the three SOD isoforms (cytosolic Cu/Zn-SOD, mitochondrial MnSOD, and extracellular EC-SOD). Due to its affinity to heparin, EC-SOD can be separated from the intracellular isoforms by passing the samples over a concanavalin A sepharose column [124]. On the other hand, applying differential centrifugation to the samples results in a mitochondrial pellet and cytosolic supernatant, which can be used to assess the separate activities of MnSOD and Cu/Zn-SOD, respectively [125, 126]. The selective measurement of MnSOD activity can also be achieved by the addition of sodium cyanide to the samples to inhibit the Cu/Zn-SOD isoform [127, 128].

*(2) Catalase*. Catalase is a ubiquitously expressed antioxidant enzyme that is responsible for the degradation of hydrogen peroxide into water and oxygen [129]. Numerous methods have been designed to assess the activity of this antioxidant in biological samples. A quantitative spectrophometric method, developed by Beers and Sizer [130] and described by Nelson and Kiesow [131] or Aebi [132], follows the breakdown of hydrogen peroxide catalyzed by catalase, by observing the decrease in ultraviolet absorbance of a hydrogen peroxide solution at *λ* = 240 *nm* as a function of time [67, 75, 77–79]. Another simple colorimetric assay based on the utilization of hydrogen peroxide by catalase using the K_2_Cr_2_O_7_/acetic acid reagent has been described by Sinha [133]. When heated in the presence of hydrogen peroxide, the dichromate in acetic acid reduces to chromic acetate, which can be measured colorimetrically at 610 nm [119]. In both these methods, the catalase activity is determined by the disappearance of hydrogen peroxide and each unit is therefore defined as the amount that degrades 1 *μ*mol of hydrogen peroxide per minute. Alternatively, the activity of the catalase enzyme can be determined using another spectrophotometric assay developed by Goth [134], which measures the stable complex formation of hydrogen peroxide with ammonium molybdate at an absorbance of 405 nm [121].

In contrast to the previously discussed assays, the method developed by Johansson and Håkan Borg [135] determines the activity of catalase using its peroxidatic function of alcohol oxidation. In this method, the formaldehyde, generated by the reaction of catalase with methanol in the presence of an optimal concentration of hydrogen peroxide, is measured spectrophotometrically at *λ* = 550 *nm* with purpald reagent (4-amino-3-hydrazino-5-mercapto-1,2,4-triazole) as a chromagen [123].

*(3) Glutathione Peroxidase*. Glutathione peroxidase (GPx) is another antioxidant enzyme that catalyzes the reduction of hydrogen peroxide and lipid peroxides to water and their corresponding lipid alcohols *via* the oxidation of reduced glutathione (GSH) into glutathione disulfide (GSSG) [136]. Its activity can be assessed by the method of Rotruck et al. [137], as described by Hafeman et al. [138], in which the samples are incubated with hydrogen peroxide in the presence of glutathione for a particular time period. The amount of utilized hydrogen peroxide is then determined by directly estimating GSH content using Ellman's reagent, 5,5′-dithiobisnitrobenzoic acid (DTNB) (discussed in Section 1) [139]. Another method developed by Kokatnur and Jelling [140] and later described by Paglia and Valentine [141] and Pleban et al. [142] relies on a similar principle, with GPx catalyzing the oxidation of glutathione by cumene hydroperoxide (for selenium-independent GPx) or hydrogen peroxide (for selenium-dependent GPx). However, in this method, the oxidized glutathione is later reduced by exogenous glutathione reductase causing the coenzyme of the reaction, NADPH, to become oxidized into NADP+. The change in the absorbance can then be read spectrophometrically at *λ* = 340 *nm* [67, 119, 123, 143].

*(4) Glutathione S-Transferase*. Glutathione S-transferases (GSTs) are members of the multigene family of isoenzymes that are ubiquitously expressed in humans. In addition to their catalytic role in conjugating GSH to a variety of harmful electrophilic compounds for detoxification, a number of GST isoenzymes reduce lipid hydroperoxides through their selenium-independent GPx activity and detoxify lipid peroxidation end-products such as 4-HNE [144, 145]. Their activity is most commonly determined by the method of Habig et al. [146], which is based on the ability of GST to conjugate 1-chloro-2,4-dinitrobenzene (CDNB) to reduced glutathione. The conjugation is accompanied by an increase in absorbance at 340 nm which can be measured spectrophometrically to directly estimate the level of GST activity in clinical samples [66, 119, 123, 143].

#### 2.3.2. Nonenzymatic Antioxidants

*(1) Glutathione*. Inside the cell, free glutathione can exist as the reduced GSH and oxidized GSSG forms, although it is primarily maintained in the former state by glutathione reductase [147]. GSH is the most abundant intracellular low-molecular-weight thiol and plays a critical role in metabolic protective functions, including hydroperoxide reduction, xenobiotic detoxification, and free radical scavenging [148]. The levels of GSH are commonly determined by the method developed by Ellman [139] and described by Beutler et al. [149], Sedlak and Lindsay [150], or Hu [151], based on the ability of the Ellman reagent, DTNB, to react with compounds containing sulfhydryl groups, yielding a mixed disulfide (GS-TNB) and 2-nitro-5-thiobenzoic acid (TNB). The levels of the latter are quantified spectrophometrically by measuring the absorbance of the anion (TNB^2-^) at 412 nm using a molar extinction coefficient of 14,150 M^−1^ cm^−1^ [143]. This method has been modified by Tietze [152] using an enzymatic recycling procedure by glutathione reductase to enhance the sensitivity of the assay and to measure instead the total glutathione content of the biological samples. In this method, the NADPH-dependent glutathione reductase of the recycling system subsequently reduces the generated GS-TNB, releasing a second TNB molecule and recycling GSH, thereby amplifying the response. In addition, any GSSG, present in the samples or formed during the reaction, is also reduced to GSH by glutathione reductase. Therefore, the glutathione concentration of the sample measured at an absorbance of 415 nm would account for both GSH and GSSG levels [75, 153].

Alternatively, the level of GSH can be determined using the GSH-400 method, based on a two-step chemical reaction followed by a spectrophotometric detection. First, 4-chloro-7-trifluoromethylbenzopyridine reacts with all mercaptans present in the sample to form substitution products (thioethers). Then, the passage through an alkaline medium gives rise to a specific *β*-elimination reaction of the thioether obtained with glutathione, leading to the formation of a chromophoric thione with an absorbance of 400 nm [83, 154].

Fluorometric assays have also been developed for glutathione analysis offering high specificity and specificity. The most frequently used probe, ortho-phthalaldehyde (OPA) or its analogue 2,3-naphthalenedicarboxaldehyde, reacts with GSH to form a highly fluorescent product with Ex/Em = 340/420 nm [155–157]. Other fluorescent probes, such as monochlorobimane (MCB) and monobromobimane (MBB), used less frequently, also form stable fluorescent adducts with GSH, which can be determined at Ex/Em = 394/490 nm. In comparison with OPA, MCB and MBB have the substantial advantage of penetrating into the cell to react directly with cellular thiols, preventing possible GSH oxidation after cell lysis and allowing analysis by flow cytometry and fluorescence microscopy [158–161].

*(2) Vitamin A*. Vitamin A refers to a group of fat-soluble retinoids (retinol, retinal, and retinyl esters) and provitamin A carotenoids (most notably *β*-carotene) that function as important dietary antioxidants, due to their ability to scavenge and directly neutralize free radicals [162]. The levels of retinoids and carotenoids are typically measured in plasma/serum or tissue samples to assess vitamin A inadequacy using atmospheric pressure chemical ionization (APCI) liquid chromatography/mass spectrometry [80, 163] or reversed-phase HPLC [164–168].

*(3) Vitamin C*. Water-soluble vitamin C (ascorbic acid), primarily found in the cytosol and extracellular fluid, plays a protective effect in reducing oxidative damage by reacting with ROS molecules such as aqueous peroxyl radicals [14, 169]. The total ascorbic acid level is commonly estimated by the method developed by Roe and Keuther [170]. This method involves the oxidation of ascorbic acid into dehydroascorbic acid by Cu^2+^, followed by its coupling with 2,4-dinitrophenylhydrazine (DNPH). The resulting derivative is then treated with strong acid leading to the production of an orange-red product, which can be measured spectrophometrically at 520 nm [171]. Ascorbic acid can also be measured using the method developed by Zannoni et al. In this method, ferric iron is reduced by ascorbic acid, producing ferrous iron, which then forms a red-colored complex with 2,2′-dipyridyl, displaying an absorbance at 520 nm [172]. It is worth noting that incubation of the samples with dithiothreitol prior to performing this assay, as suggested by Masato, would reduce dehydroascorbic acid into ascorbic acid, enabling the determination of total ascorbic acid in clinical samples [173]. Alternatively, the levels of vitamin C can be determined using reversed-phase HPLC [174–177].

*(4) Vitamin E*. Vitamin E (*α*-tocopherol) is a lipid-soluble vitamin which acts as a lipid peroxyl radical scavenger, preventing lipid peroxidation chain reactions in the cell membranes [14, 169].

Its level can be measured by the method of Emmerie and Engel [178], later described by Hashim and Schuttringer [179] and Baker et al. [180], and is based on the reduction of ferric ions to ferrous ions by *α*-tocopherol. Similar to the method by Zannoni et al. (discussed in Section 3), the ferrous ions are then coupled with 2,2′-dipyridyl, which can be detected colorimetrically at 520 nm [119]. The method of Desai also involves the reduction of ferric ions by vitamin E, but the formation of a pink-colored complex is achieved with batophenanthroline orthophosphoric acid, and the absorbance is read at 536 nm [181].

Vitamin E levels in serum can also be estimated using fluorometry by the method of Hansen and Warwick [182]. Following the precipitation of serum proteins by alcohol, vitamin E can be extracted into hexane level and quantified at *λ*_excitation_ = 295 nm and *λ*_emission_ = 340 nm [183]. Alternatively, tocopherols can be analyzed by gas chromatography-mass spectrometry (GC-MS) on a SPB1 column using a selected ion monitoring technique [77–79] or by reversed-phase HPLC [164–166, 168].

#### 2.3.3. Total Antioxidant Capacity

Until recently, investigating the status of antioxidants has been carried out by measuring the levels or activities of each separately. However, measurement of the overall effect of antioxidants can be quite useful in assessing the oxidative state in clinical samples due to the various interactions between the different antioxidants [184]. Several methods that are less time-consuming and labor-intensive have therefore been developed to determine the total antioxidant status (TAS) in clinical samples.

The 2,2-diphenyl-1-picryl-hydrazyl (DPPH) reduction assay is a method that uses free radical traps to assess the antioxidant capacity of the samples. DPPH is a stable free radical due to the delocalization of the spare electron over the molecule as a whole, with a deep violet color characterized by an absorption band at 520 nm. When DPPH is mixed with an antiradical compound that can neutralize it, it becomes colorless. Therefore, the decrease in optical density of DPPH radicals is monitored to evaluate the antioxidant potential of the samples [185–187].

The 2,2′-azino-bis (3-ethylbenz-thiazoline-6-sulfonic acid) (ABTS) assay, developed by Erel [188, 189], similarly uses a strongly colored stable radical compound to evaluate the antioxidant state of samples. Here, ABTS is first oxidized by metmyoglobin and hydrogen peroxide into its radical cation form (ABTS•+), a blue-green chromophore with an absorption at 750 nm. When antioxidants are added, ABTS•+ is reduced to ABTS and becomes decolorized again. Therefore, this method also follows the discoloration of the stable radical spectrophotometrically to measure the relative antioxidant ability of the samples. This assay is often referred to as Trolox equivalent antioxidant capacity (TEAC) method, because the reaction rate is commonly calibrated with a water-soluble analogue of vitamin E, Trolox (6-hydroxy-2,5,7,8-tetramethylchroman-2-carboxylic acid), as an antioxidant standard. The ABTS assay has an advantage over other techniques in that it is freely soluble in both organic and aqueous solvents so it is applicable for both hydrophilic and lipophilic antioxidants [90, 185, 190–193].

The total radical-trapping antioxidant parameter (TRAP) assay is another free radical trapping method which has been widely applied to evaluate the antioxidant capacity particularly in plasma. In this assay, thermal decomposition of the water-soluble azo compound 2,2′-azobis(2-methylpropionamidine) dihydrochloride (ABAP) generates peroxyl radicals at a known steady rate, which is monitored through a linear decrease in R-phycoerythrin (R-PE) fluorescence over time using a luminescence spectrometer (*λ*_excitation_ = 495 nm and *λ*_emission_ = 575 nm). When the sample is added to the reaction mixture, the antioxidants provide protection for the fluorescence decay of R-PE. The length of the lag phase is therefore used to directly estimate the total antioxidant capacity [75, 185, 194, 195]. This method is relatively more complex and time-consuming than the others are.

The ferric-reducing antioxidant power (FRAP) assay is a simpler and faster method to assess the antioxidative potential of plasma; however, this method cannot detect antioxidants that act by radical quenching. The colorimetric assay is based on the ability of antioxidants to reduce the ferric tripyridyltriazine (Fe^3+^-TPTZ) complex to the ferrous form at low pH. The end-product (Fe^2+^-TPTZ) has an intense blue color with absorption at 593 nm which can be monitored using a diode-array spectrophotometer to estimate the antioxidant capacity of the samples [45, 185, 196].

The biological antioxidant potential (BAP) test is another assay based on the ability of antioxidants to reduce ferric ions to ferrous ions. However, in this method, ferric ions are bound to a chromogenic thiocyanate derivative substrate, which becomes decolorized when the ferric ions are reduced to ferrous ions by the antioxidants of the serum sample. This reduction is therefore quantified to estimate the antioxidant capacity of the sample by measuring the absorbance change at 505 nm [44, 197].

As for the method developed by Koracevic et al., a standardized solution of the Fe-EDTA complex reacts with hydrogen peroxide by a Fenton-type reaction leading to the formation of hydroxyl radicals which degrade sodium benzoate solution resulting in the release of colored TBARS. Addition of antioxidants inhibits the production of TBARS, and the inhibition of color development is detected spectrophotometrically at 532 nm to estimate the total antioxidant status in the clinical samples [198].

## 3. Oxidative Stress in Clinical Samples of Cancer Patients

ROS is a contributing factor in the natural process of aging as well as in various pathological diseases including cancer. Oxidative stress has been reported in almost all types of cancers, promoting many aspects of tumor development and progression [199]. During the process of carcinogenesis, an increase in ROS levels can occur due to elevated metabolic activity, oncogene activation, increased cellular receptor signaling, or mitochondrial dysfunction. Overproduction of ROS can also be induced exogenously by carcinogenic insults such as cigarette smoke, heavy metals, ionizing radiation, and asbestos [199–202]. Alternatively, oxidative stress observed in cancer cells can arise from low levels or inactivation of antioxidant defense mechanisms as a result of mutations in tumor suppressor genes. For instance, mutant BRCA1 and p53 have been shown to attenuate the activation and function of nuclear factor (erythroid-derived 2)-like 2 (Nrf2), a transcription factor that stimulates a stress response pathway by inducing expression of ROS-detoxifying enzymes [203–205].

The free radicals, generated during oxidative stress conditions, can consequently act as secondary messengers in intracellular signaling pathways involved in cell cycle progression and proliferation, cell survival and apoptosis, cell migration and angiogenesis, tissue invasion and metastasis, and tumor stemness, thereby affecting all characters of oncogenic phenotype of cancer cells [199, 200]. In addition to these oxidative stress-mediated signaling events, high levels of ROS can also lead to nonspecific damage of macromolecules such as nucleic acids, proteins, and lipids, often creating more free radicals, and triggering a chain of destruction, to promote oncogenic transformation [206]. ROS can cause individual DNA base changes or gross chromosomal alterations, thereby inducing mutagenesis and genomic instability. The cells harboring DNA mutations typically undergo apoptosis if they are unable to completely repair these DNA lesions; however, under certain circumstances, these cells escape programmed cell death, giving rise to transformed progeny [14, 200, 207–209]. The mutagenic effects of ROS are not limited to DNA damage, but can also involve an attack of ROS molecules on proteins and lipids. For instance, oxidative damage to the cells' lipids initiates lipid peroxidation, leading to the generation of a range of mutagenic products that could alter cellular functions and enhance cancer initiation or progression [210, 211]. On the other hand, ROS-induced protein damage to DNA repair enzymes has been suggested as an explanation for the increased susceptibility of mutations which contribute to the process of carcinogenesis [212].

Identification of oxidative stress biomarkers in clinical samples of cancer patients and defining their roles in cancer initiation and progression holds great promise in promoting the development of targeted therapeutic approaches and diagnostic strategies evaluating disease status. While literature records of direct measurement of ROS levels in clinical samples are limited, significant amount of data do exist regarding oxidative damage to the cell's biomolecules (DNA, lipids, and proteins) as well as on enzymatic, nonenzymatic, and total antioxidant status in clinical samples of cancer patients. In the tables below, we summarize some of these findings in 10 different types of cancer.  Table 1 outlines findings regarding oxidative damage to DNA, lipids, and proteins in clinical samples of cancer patients, while Tables 2, 3, and 4 summarize data related to enzymatic antioxidant activities, nonenzymatic antioxidant levels, and total antioxidant status, respectively. We have particularly selected publications that report quantitative values of the oxidant/antioxidant markers.

## 4. Conclusion

Oxidative stress is implicated in the natural process of aging as well as in a variety of disease states. A detailed understanding regarding the link between oxidative stress and pathogenesis can be exploited to assess disease status as well as to develop preventive and therapeutic strategies in humans. In this review, three approaches were suggested to assess oxidative stress in clinical samples: (1) direct measurement of ROS levels, (2) detection of the resulting oxidative damage to biomolecules (DNA, lipids, and proteins), and (3) determination of antioxidant status (enzymatic antioxidant activities, nonenzymatic antioxidant levels, or total antioxidant capacity).

Direct ROS determination is a valuable and promising oxidative stress biomarker that can reflect on disease status. However, as we noted earlier, their measurement in biological systems is a complex task given the short half-life and high reactivity of these species. On the other hand, “footprints” of oxidative stress are extremely stable and provide a more reliable approach to evaluate oxidative stress in clinical samples. While some of these modifications only reflect the local degree of oxidative stress, others have a direct effect on the function of target molecules. This functional significance or the causal role of oxidative modifications further highlights the clinical applicability of these oxidative stress markers. However, sample processing should be performed with caution to ensure their stability and to avoid the possibility that biomolecules may become oxidatively damaged during their isolation. As for the correlation of the antioxidant status to the state of oxidative stress in clinical samples, the measurement of individual antioxidant levels/activities could yield conflicting results. For instance, some papers report low antioxidant status in cancer samples, explaining it as a loss of their protective capacity due to high oxidative stress, while others interpret the findings of high levels/activities of the antioxidants as an adaptive response mechanism to detoxify oxidative stress-related harmful metabolites. To overcome such biases, it is advisable to determine the total antioxidant status by evaluating all antioxidants simultaneously without excluding their interactions with each other.

The choice of the oxidative stress biomarkers and the methods used to measure them in order to assess oxidative status in clinical samples should be decided based on the aim of the study and its design, as well as on the clinical relevance in the selected subjects. No single parameter has yet been recommended as a gold standard for defining redox status in clinical samples. Furthermore, the individual markers described above only partially reflect on oxidative status. Therefore, an integrative approach examining both pro- and antioxidant reactions has been recently suggested to obtain a comprehensive score with higher sensitivity to physiological and pathological alternations. Global redox status indexes such as OXY-SCORE or oxidative-INDEX, computed by subtracting the antioxidant capacity from ROS levels/ROS-induced damage, or oxidative stress index (OSI), which is the ratio of total oxidant status to total antioxidant status, reflect simultaneously on oxidative and antioxidant status in clinical samples and provide a better and more powerful index in the evaluation of overall oxidative stress in clinical samples and the establishment of a definitive relationship between oxidative stress and disease status [280–283].

As a final note, to our knowledge, no adequate comparison has yet been performed between different biomarkers and the methodologies used to measure them, making it difficult if not impossible to make a reliable comparison of findings from different groups. A critical evaluation and adaptation of proposed methodologies available in the literature should therefore be undertaken prior to carrying out a proposed study, so as to enable the investigators to choose the most suitable procedure for each chosen biomarker. In addition, such a comparison will enable careful meta-analysis of multiple scientific studies related to oxidative stress.

## Figures and Tables

**Table 1:** Oxidative damage to DNA, lipids, and proteins in clinical samples of cancer patients.

Type of cancer	Oxidative damage		
	DNA	Lipid	Protein
Bladder cancer	*Urinary 8-OHdG (ELISA)*: 8.12 ng/g creatinine compared to 4.13 ng/g in controls (*p* = 0.019) [213] *Serum 8-OHdG (ELISA)*: 0.24 ng/mL *Urinary 8-OHdG (ELISA)*: 12.2 ng/mL [102] *Urinary 8-OHdG (ELISA)*: 70.5 ± 38.2 ng/mg creatinine compared to 36.1 ± 24.5 ng/mg in controls (*p* < 0.001) [100]	*Urinary MDA (TBARS)*: 9.54 *μ*mol/g creatinine compared to 6.76 *μ*mol/g in controls (*p* = 0.024) [213] *Serum MDA (Yoshioka's TBARS)*: 13.91 ± 8.59 nmol/mL compared to 2.12 ± 0.78 nmol/mL in controls (*p* < 0.001) [183] *Tissue MDA (TBARS)*: 4.29 ± 3.2 *μ*mol/mg protein compared to 2.3 ± 0.6 *μ*mol/mg in controls (ns) [214]	*Plasma PC content (DNPH)*: 0.5 mg/mg protein compared to 0.38 mg/mg in controls (*p* < 0.001) [Figure 3(b)] [215]

Breast cancer	*Tissue 8-OHdG (HPLC)*: 2.07 ± 0.95 per 10^5^ dG compared to 1.34 ± 0.46 in corresponding noncancerous tissues (*p* < 0.0001) [216] *Tissue 8-OHdG (HPLC)*: 10.7 ± 15.5 per 10^5^dG compared to 6.3 ± 6.8 in controls (*p* = 0.035) *Tissue 8-OHdG (IHC)*: 3.9 ± 7.2 signal intensity compared to 1.1 ± 1.4 in controls (*p* = 0.008) *Tissue hOGG1 (IHC)*: 3.34 ± 1.95 signal intensity compared to 2.27 ± 1.96 in controls (*p* = 0.008) [108]	*Serum MDA (TBARS)*: 6 *μ*mol/L compared to 2 *μ*mol/L in controls (*p* < 0.05) [Figure 1(a)] [217] *Plasma MDA (Yagi's TBARS)*: 5.07 ± 0.53 nmol/mL compared to 2.54 ± 0.38 nmol/mL in controls (*p* < 0.05) *Erythrocyte MDA (Donnan's TBARS)*: 2.95 ± 0.76 pmol/mg Hg compared to 2.23 ± 0.47 pmol/mg in controls (*p* < 0.05) [218] *Tissue MDA (Ohkawa's TBARS)*: 5.92 ± 1.41 nmol/mg protein compared to 4.09 ± 1.25 nmol/mg in adjacent normal tissues (*p* < 0.001) *Tissue LOOH (FOX method)*: 0.57 ± 0.16 mmol/g tissue compared to 0.41 ± 0.12 mmol/g in adjacent normal tissues (*p* < 0.001) *Tissue CD (Suryanarayana Rao and Recknagel)*: 0.55 ± 0.17 mg/g tissue compared to 0.39 ± 0.11 in adjacent normal tissues (*p* < 0.001) [219] *Blood (TBARS)*: 26.14 nmol/mL compared to 15.83 nmol/mL in controls (*p* < 0.01) [220]	*Tissue PC content (ELISA)*: 3.5 nmol/mg protein lysate compared to 2.3 nmol/mg in adjacent normal tissues (*p* < 0.05) [Figure 1(a)] [221]

Cervical cancer	*Cellular 8-OHdG (IHC)*: 64.5 ± 17.4 (low grade) and 91.8 ± 22.5 signal intensity (high grade) compared to 50.2 ± 14.2 in controls (*p* = 0.17 and *p* < 0.001) [222] *Urinary 8-OHdG (ELISA)*: 8.94 ± 1.70 ng/mg creatinine compared to 10.27 ± 2.60 ng/mg in controls (ns) [223]	*Plasma MDA (Yagi's TBARS)*: 6.5 nmol/mg Hg compared to 3.5 nmol/mg in controls (*p* < 0.001) [Figure 1] *Plasma CD (Suryanarayana Rao and Recknagel)*: 1.75 *μ*mol/mg Hg compared to 0.75 *μ*mol/mg in controls (*p* < 0.001) [Figure 1] [224] *Plasma MDA (Ohkawa's TBARS)*: 6.2 nmol/mg Hb compared to 3.1 nmol/mg in controls (*p* < 0.0001) [Figure 2] *Erythrocyte MDA (Donnan's TBARS)*: 5.1 nmol/mg Hg compared to 3 nmol/mg in controls (*p* < 0.0001) [Figure 5] [119] *Plasma MDA (HPLC)*: 13.61 ± 0.73 nmol/mL compared to 9.85 ± 0.69 nmol/mL in controls (*p* < 0.05) [223] *Plasma LOOH (FOX method)*: 0.84 ± 0.33 mmol/L compared to 0.36 ± 0.17 mmol/L in controls (*p* < 0.001) [225]	*Plasma PC content (Levine's DNPH)*: 1.47 ± 0.54 nmol/mg compared to 0.80 ± 0.39 nmol/mg in controls (*p* < 0.001) [225]

Colorectal cancer	*Tissue 8-OHdG (IHC)*: 229.1 ± 20.0 staining intensity compared to 113.3 ± 7.1 in corresponding normal epithelial cells (*p* < 0.001) *Tissue 8-OHdG (HPLC)*: 2.53 ± 0.15 per 10^5^ dG compared to 1.62 ± 0.13 in corresponding surrounding tissues (*p* < 0.005) [94] *Tissue 8-OHdG (HPLC)*: 49 [range: 23-114] per 10^6^ dG compared to 21 [range: 9-39] per 10^6^ dG in corresponding normal mucosa (*p* = 0.012) [96] *Tissue 8-OHdG (HPLC)*: 1.34 ± 0.11 per 10^5^dG compared to 0.64 ± 0.05 per 10^5^dG in nontumorous counterparts (*p* < 0.001) *Tissue 8-OHdG lyase activity (Yamamoto)*: 36.16 ± 1.79% compared to 19.72 ± 1.13 in nontumorous counterparts (*p* < 0.001) *Tissue hOGG1 transcripts (quantitative RT-PCR)*: 30.58 ± 3.21 × 10^5^ copies/*μ*g RNA compared to 4.55 ± 0.54 × 10^5^ copies/*μ*g in nontumorous counterparts (*p* < 0.001) [95] *Tissue 8-OHdG (HPLC)*: 2.4 ± 1.1 per 10^5^ dG compared to 1.6 ± 0.6 per 10^5^ dG in normal mucosa (*p* = 0.007) *Tissue hOGG (HPLC)*: 4.3 ± 1.3 pmol of OHdG released from DNA/1 h compared to 2.7 ± 1.2 in normal mucosa (*p* = 0.022) [109] *Blood 8-OHdG (HPLC)*: 13.76 ± 7.19 per 10^6^dG compared to 9.57 ± 3.95 per 10^6^dG in controls (*p* = 0.0034) [174]	*Serum oxidized-LDL (ELISA)*: 39.2 U/L compared to 36.2 U/L in controls (*p* = 0.045) [226] *Tissue MDA (HPLC)*: 1.7 ± 0.39 (stage II), 2.25 ± 0.43 (stage III), and 4.04 ± 0.47 nmol/g tissue (stage IV) compared to 1.39 ± 0.15 in normal colon mucosa (*p* < 0.001) *Tissue 4-HNE (HPLC)*: 0.37 ± 0.07 (stage II), 0.45 ± 0.09 (stage III), and 0.52 ± 0.11 nmol/g tissue (stage IV) compared to 0.29 ± 0.03 in normal colon mucosa (*p* < 0.001) [83] *Tissue MDA (Ohkawa's TBARS)*: 81.1 ± 17.8 nmol/mg protein compared to 69.8 ± 12.8 in normal mucosa (*p* = 0.046) [109] *Tissue MDA (Beuge and Aust's TBARS)*: 0.86 ± 0.10 nmol/mg tissue compared to 0.54 ± 0.08 in normal samples (*p* < 0.01) [227]	*Plasma PC content (ELISA)*: 101.9 ± 27.4 pmol/mg plasma compared to 97.0 ± 16.8 pmol/mg plasma in controls (*p* < 0.001) [228]

Lung cancer	*Tissue TG (IHC)*: 72 ± 8.0 and 79 ± 4.9 percent positive rate in nonlesion sites of never smokers and current smokers, respectively [44]	*Plasma MDA (TBARS)*: 0.78 ± 0.5 nmol/mL compared to 1.23 ± 1.03 in smoker controls and 1.19 ± 1.09 in nonsmoker controls (ns) [120] *Serum MDA (Ohkawa's TBARS)*: 3.8 ± 2.5 nmol/mL compared to s 1.4 ± 0.8 in healthy smoker controls (*p* < 0.05) [229]	*Plasma PC content (Levine's DNPH)* :1*E* − 05 ± 5*E* − 06 nmol/mg protein compared to 8*E* − 06 ± 5*E* − 06 in smoker controls and 7*E* − 06 ± 9*E* − 06 in nonsmoker controls (*p* = 0.003) [120]

Liver cancer	*Tissue 8-OHdG (IHC)*: 8.605 staining intensity compared to 4.845 in nonalcoholic steatohepatitis controls (*p* = 0.003) [105] *Tissue 8-OHdG (HPLC)*: 52 fmol/*μ*g DNA compared to 129 fmol/*μ*g in adjacent normal tissues (*p* = 0.003) [230]	*Plasma MDA (TBARS)*: 1.01 ± 0.28 *μ*mol/L compared to 0.97 ± 0.88 *μ*mol/L in corresponding post-resection samples (*p* < 0.05) *Tissue MDA (TBARS)*: 0.46 ± 0.50 *μ*mol/g protein compared to 0.85 ± 0.42 *μ*mol/g in adjacent normal tissues (*p* < 0.05) *Plasma oxidized-LDL (ELISA)*: 37.64 ± 8.00 U/L compared to 33.72 ± 8.71 U/L in corresponding post-resection samples (*p* < 0.05) [90] *Tissue MDA (TBARS)*: 0.49 nmol/mg protein compared to 0.71 nmol/mg in adjacent normal tissues (*p* = 0.001) [230] *Tissue MDA (Ohkawa's TBARS)*: 0.154 ± 0.06 nmol/mg protein compared to 0.087 ± 0.038 nmol/mg in adjacent normal tissues (*p* < 0.05) [143] *Plasma MDA (HPLC)*: 3.26 ± 0.46 nmol/mL (stage I/II) and 5.83 ± 0.68 nmol/mL (stage III/IV) compared to 1.10 ± 0.23 in controls (*p* < 0.05) [231] *Urine 8-iso-PGF2α (LC-MS)*: 0.92 pmol/mg creatinine compared to 0.8 pmol/mg in controls (*p* < 0.001) [232]	*Plasma PC content (DNPH)*: 0.3 nmol/mg protein compared to 0.2 nmol/mg in controls (*p* = 0.0083) [Figure 1(a)] [233]

Ovarian cancer	*Serum 8-OHdG (ELISA)*: 198.6 ± 80.2 pg/mL [101] *Serum 8-OHdG (ELISA)*: 3.22 ± 0.49 ng/mL [234] *Tissue 8-OHdG (ELISA)*: 27.8 ± 8.98 per 10^6^ dG compared to 18.8 ± 5.2 per 10^6^ dG in adjacent normal tissues (*p* = 0.02) [235]	*Tissue MDA (TBARS)*: 0.65 *μ*M compared to 0.15 *μ*M in controls (*p* = 0.009) [Figure 2] [33] *Serum MDA (Buege and Aust's TBARS)*: 4.18 ± 0.80 nmol/mL (stage II) and 6.23 ± 1.37 nmol/mL (stage IV) compared to 2.51 ± 0.60 in controls (*p* < 0.001) [236] *Serum MDA (TBARS)*: 8.7 ± 3.0 nmol/L compared to 6.7 ± 2.7 nmol/L in controls (*p* = 0.002) [237] *Erythrocytes MDA (TBARS)*: 5.9 ± 0.6 nmol/g Hb compared to 5.3 ± 0.3 nmol/g in controls (*p* < 0.001) [238]	*Tissue PC content (Mesquita's DNPH)*: 18 nmol/mg total protein compared to 5.5 nmol/mg in controls (*p* = 0.009) [Figure 3] [33] *Tissue PC content (Levine's DNPH)*: 10-14 nmol/mg protein compared to 4 nmol/mg in normal surrounding samples (*p* < 0.05) [Figure 1] [56]

Prostate cancer	*Urinary 8-OHdG (ELISA)*: 58.8 ± 43.4 ng/mg creatinine compared to 36.1 ± 24.5 ng/mg in controls (*p* = 0.021) [100] *Urinary 8-OHdG (ELISA)*: 20.8 ± 8.9 ng/mg creatinine compared to 16.1 ± 7.8 ng/mg in controls (*p* < 0.05) [239]	*Plasma MDA (TBARS)*: 10.5 nmol/mL compared to 7.5 nmol/mL in controls (*p* < 0.05) [Figure 1(a)] [171] *Serum MDA (Yoshioka's TBARS)*: 16.98 ± 6.66 nmol/mL compared to 4.45 ± 1.65 nmol/mL in controls (*p* < 0.005) [240] *Erythrocytes MDA (TBARS)*: 43.0 ± 19.7 nmol/g Hb compared to 24.1 ± 8.8 nmol/g in controls (*p* < 0.001) *Plasma MDA (TBARS)*: 44.6 ± 19.7 × 10^−2^ nmol/mL compared to 28.5 ± 8.8 × 10^−2^ nmol/mL in controls (*p* < 0.001) [67]	*Serum PC (Levine's DNPH)*: 1.06 nmol/mg protein compared to 0.88 nmol/mg in controls (*p* < 0.05) [Figure 1(b)] [171]

Skin cancer	*Urinary 8-OHdG (ELISA)*: 110.08 ± 30.09 ng/mg creatinine compared to 61.92 ± 17.35 ng/mg in controls (*p* < 0.001) *Tissue 8-OHdG (IHC)*: 2.88 ± 0.30 IRS compared to 2.30 ± 0.67 in controls and 2.27 ± 0.72 in adjacent epidermis (*p* < 0.001) *Tissue hOGG1 (IHC)*: 1.77 ± 0.40 IRS compared to 2.56 ± 0.47 in controls and 2.53 ± 0.47 in adjacent epidermis (*p* < 0.001) [107]	*Plasma MDA (TBARS)*: 2.367 *μ*M compared to 1.860 *μ*M in controls (*p* < 0.0001) [241] *Cellular PUFAs (GC-MS)*: 11.8 ± 4.1% arachidonic acid/total fatty acids compared to 4.67 ± 0.9% in normal melanocytes (*p* < 0.001) [79] *Plasma MDA (HPLC)*: 281.2 ± 17.36 nM (T1/T2) and 377.9 ± 36.15 nM (T3/T4) compared to 191.7 ± 15.01 nM in controls (*p* < 0.0001) [75] *Serum MDA (TBARS)*: 65 *μ*mol/L compared to 52 *μ*Mol/L in controls (*p* < 0.001) [Figure 5] [242]	N/A

Thyroid cancer	*Serum 8-OHdG (ELISA)* :4.98 ± 2.56 ng/mL compared to 3.72 ± 1.17 in controls (*p* < 0.001) [243]	*Tissue MDA (Uchiyama and Mihara's TBARS)*: 233 ± 36 nM/g tissue (follicular carcinoma) and 239 ± 29 nmol/g (papillary carcinoma) compared to 141 ± 17 nmol/g in controls (*p* < 0.05) [244] *Tissue MDA (Uchiyama and Mihara's TBARS)*: 127.00 ± 2.82 nmol/g tissue compared to 87.75 ± 4.71 nmol/g in adjacent tissue (*p* = 0.01) [245] *Blood MDA (Uchiyama and Mihara's TBARS)*: 3.59 ± 0.1 nmol/mL compared to 1.23 ± 0.1 nmol/mL in controls (*p* < 0.05) *Blood LOOH (FOX method)*: 4.94 ± 0.83 *μ*M/mL compared to 1.7 ± 0.25 *μ*M/mL in controls (*p* < 0.05) [246] *Serum LOOH (FOX method)*: 14.71 ± 8.68 *μ*mol/L compared to 6.51 ± 3.01 *μ*mol/L in controls (*p* < 0.001) [243] *Plasma MDA (Yagi's TBARS)*: 6.57 ± 0.7 nmol/mL compared to 2.89 ± 0.30 nmol/mL in controls (*p* < 0.001) *Erythrocytes MDA (Donnan's TBARS)*: 6.63 ± 0.6 pmol/mg Hb compared to 3.20 ± 0.32 pmol/mg in controls (*p* < 0.001) [247]	*Blood AOPP (Witko-Sarsat)*: 271.14 ± 16.40 *μ*mol/mL compared to 61.59 ± 16.42 in controls (*p* < 0.05) [246] *Blood PC content (Levine's DNPH)*: 6.21 ± 0.63 nmol/mg compared to 4.32 ± 0.24 in controls (*p* < 0.05) [248]

**Table 2:** Enzymatic antioxidant activities in clinical samples of cancer patients.

Type of cancer	Enzymatic antioxidant activities			
	SOD	Catalase	GPx	GST
Bladder cancer	*In whole blood (Oyanagui/Sun et al.)*: 28.49 ± 14.03 U/mL compared to 194 ± 28.48 U/mL in controls (*p* < 0.001) [183] *In serum (HPLC)*: 149.140 ± 29.65 U/mL compared to 201 ± 31.4 U/mL in controls (*p* < 0.001) [249] *In plasma (Oyanagui)*: 24.9 ± 1.64 U/mL compared to 21.4 ± 2.08 U/mL in controls (ns) [250]	*In serum (Aebi)*: 1.85 ± 0.07 U/L compared to 1.15 ± 0.04 U/L in controls (*p* < 0.001) [251] *In serum (HPLC)*: 10.4430 ± 2.47984 U/L compared to 20 ± 4.3 U/L in controls (*p* < 0.001) [249]	*In whole blood (Paglia and Valentine)*: 1693.09 ± 544.01 U/L compared to 6906 ± 847 U/L in controls (*p* < 0.001) [183] *In serum (HPLC)*: 131.0076 ± 14.46 U/L compared to 170 ± 28 U/L in controls (*p* < 0.001) [249]	*In tissue (Habig's CDNB)*: 257.3 ± 116.9 U/mg protein compared to 68.6 ± 31.0 U/mg in surrounding normal uroepithelium (*p* < 0.001) [252]

Breast cancer	*In tissue (Kakkar)*: 27.57 ± 5.08 U/mg protein compared to 19.64 ± 5.1 U/mg in adjacent normal tissues (*p* < 0.001) [219] *In serum (Oyanagui/Sun et al.)*: 500 U/g protein compared to 750 U/g protein in controls (*p* = 0.05) [217] *In erythrocytes (Kakkar)*: 2.05 ± 0.57 U/mg Hg compared to 3.22 ± 0.72 U/mg in controls (*p* < 0.05) [218] *In tissue (Oyanagui/Sun et al.)*: 20 U/mg compared to 38 U/mg in adjacent healthy tissues [Figure 4(a)] [221] *In blood (Marklund and Marklund)*: 0.12 U/gm Hg compared to 0.16 U/gm Hg in controls (*p* < 0.01) [220]	*In tissue (Sinha)*: 14.66 ± 4.1 *μ*mol H_2_O_2_ utilized/s/mg protein compared to 8.17 ± 3.14 *μ*mol/s/mg in adjacent normal tissues (*p* < 0.001) [219] *In erythrocytes (Sinha)*: 0.73 ± 0.3 *μ*mol H_2_O_2_ utilized/s/mg Hg compared to 1.45 ± 0.65 *μ*mol/s/mg in controls (*p* < 0.05) [218]	*In tissue (Rotruck et al.)*: 22.41 ± 5.87 *μ*mol GSH utilized/min/mg protein compared to 15.58 ± 5.11 *μ*mol/min/mg in adjacent normal tissues (*p* < 0.001) [219] *In serum (Paglia and Valentine)*: 90 U/g protein compared to 135 U/g in controls (*p* = 0.05) [217] *In erythrocytes (Rotruck et al.)*: 7.3 ± 0.85 *μ*mol GSH utilized/min/g Hb compared to 10.03 ± 0.89 *μ*mol/min/g in controls (*p* < 0.05) [218]	*In erythrocytes (Habig's CDNB)*: 1.14 ± 0.3987 *μ*mol CDNB-GSH conjugate formed/min/mg Hb compared to 1.65 ± 0.39 *μ*mol/min/mg in control (*p* < 0.05) [218]

Cervical cancer	*In plasma (Kakkar)*: 1 U/min/mg Hb compared to 2.5 U/min/mg in controls (*p* < 0.001) [Figure 2] [224] *In plasma (Sun et al.)*: 0.9 U/min/mg Hb compared to 2.2 U/min/mg in controls (*p* < 0.0001) [Figure 2] *In erythrocytes (Sun et al.)*: 1.5 U/min/mg Hb compared to 2.1 U/min/mg in controls (*p* < 0.0001) [Figure 5] [119] *In blood (Beyer and Fridovich)*: 1.45 ± 0.11 U/mg Hb compared to 1.84 ± 0.09 U/mg in controls (*p* < 0.05) [223]	*In erythrocytes (Sinha)*: 1.4 *μ*mol H_2_O_2_ utilized/min compared to 2 *μ*mol/min in controls (*p* < 0.0001) [Figure 5] [119] *In blood (Aebi)*: 1.26 ± 0.05 U/mg Hb compared to 1.69 ± 0.07 U/mg in controls (*p* < 0.05) [223]	*In plasma (Rotruck et al.)*: 8 *μ*mol H_2_O_2_ utilized/min/g Hb compared to 12 *μ*mol/min/g in controls (*p* < 0.001) [Figure 2] [224] *In plasma (Paglia and Valentine)*: 0.3 U/min/mg Hb compared to 0.5 U/min/mg in controls (*p* < 0.0001) [Figure 4] *In erythrocytes (Paglia and Valentine)*: 20 U/min/mg Hb compared to 25 U/min/mg in controls (*p* < 0.0001) [Figure 6] [119] *In blood (Paglia and Valentine)*: 9.16 ± 0.46 mU/mg Hb compared to 6.06 ± 0.52 mU/mg in controls (*p* < 0.05) [223]	*In plasma (Habig's CDNB)*: 1.7 *μ*mol CDNB-GSH conjugate formed/min/mg Hb compared to 2.4 *μ*mol/min/mg in controls (*p* < 0.001) [Figure 2] [224] *In plasma (Habig's CDNB)*: 1.7 U/min/mg Hb compared to 2.1 U/min/mg in controls (*p* < 0.0001) [Figure 4] [119]

Colorectal cancer	*In tissue (Cu/Zn-SOD—Misra and Fridovich)*: 237 ± 42 (stage I), 289 ± 47 (stage II), and 421 ± 58 U/g tissue (stage III) compared to 117 ± 25 in normal colon mucosa (*p* < 0.001) [83] *In tissue (Misra and Fridovich)*: 4.9 ± 2.2 U/mg protein compared to 7.5 ± 2.6 U/mg in normal mucosa (*p* = 0.002) [109] *In tissue (Oyanagui/Sun et al.)*: 88.9 ± 40.69 U/g protein compared to 58.4 ± 29.23 U/g in corresponding distal margin (*p* < 0.0001) [123]	*In tissue (Aebi)*: 76 ± 14 (stage I), 57 ± 16 (stage II), and 33 ± 18 U/g tissue (stage III) compared to 84 ± 17 U/g in normal colon mucosa (*p* < 0.001) [83] *In tissue (Beers and Sizer)*: 19.3 ± 7.4 U/mg protein compared to 24.8 ± 6.2 U/mg in normal mucosa (*p* = 0.004) [109] *In tissue (Johansson and Borg)*: 114.2 ± 63.48 U/g protein compared to 103.4 ± 53.13 U/g in corresponding distal margin (ns) [123] *In tissue (Beers and Sizer)*: 6.58 ± 1.5 U/g tissue compared to 3.94 ± 1.1 U/g in normal samples (*p* < 0.01) [227]	*In tissue (Paglia and Valentine)*:1854 ± 552 (stage I), 1987 ± 699 (stage II), and 2467 ± 368 U/g tissue (stage III) compared to 1723 ± 189 in normal colon mucosa (*p* < 0.001) [83] *In tissue (Paglia and Valentine)*: 54.5 ± 66.96 U/g protein compared to 22.8 ± 23.99 U/g in corresponding distal margin (*p* = 0.004) [123] *In tissue (Kokatnur and Jelling)*: 5.75 U/g tissue compared to 7.5 U/g in normal samples (*p* < 0.05) [Figure 2(a)] [227] *In erythrocytes (Pleban et al.)*: 7.46 ± 0.80 U/g Hb compared to 12.80 ± 0.88 U/g in controls (*p* < 0.05) [253]	*In tissue (Habig's CDNB)*: 31.0 ± 22.11 U/g protein compared to 42.5 ± 23.45 U/g in corresponding distal margin (*p* = 0.021) [123] *In erythrocytes (Habig's CDNB)*: 3.61 ± 0.99 U/g Hb compared to 0.82 ± 0.08 U/g in controls (*p* < 0.05) [253]

Lung cancer	*In plasma (MnSOD—Oyanagio/Sun et al.)*: 0.91 ± 0.85 U/mg protein compared to 5.11 ± 4.34 in smoker controls and 6.17 ± 5.54 in nonsmoker controls (*p* ≤ 0.001) [120] *In tissue (Sun et al.)*: 1.42 ± 0.24 U/mg protein compared to 3.13 ± 0.51 in adjacent normal tissues (*p* < 0.01) [121] *In serum (Oyanagui/Sun et al.)*: 1.60 U/mL compared to 1.91 U/mL (*p* < 0.01) [254]	*In tissue (Goth)*: 33.53 ± 6.09 U/mg protein compared to 71.33 ± 14.38 U/mg in adjacent normal tissues (*p* < 0.01) [121] *In serum (Johansson and Borg):* 31.1 nmol/min/mL compared to 39.73 nmol/min/mL (ns) [254]	*In serum (GPX3 - ELISA)*: 10.1 ± 5.0 *μ*g/mL compared to 13.0 ± 5.8 *μ*g/mL in controls (*p* < 0.001) [255]	*In plasma (Habig's CDNB)*: 19.94 ± 0.73 IU/L compared to 3.64 ± 0.17 in controls (*p* < 0.001) [256] *In tissue (Habig's CDNB)*: 1.72 ± 0.89 U/mg protein compared to 1.12 ± 0.43 U/mg in adjacent normal tissue (*p* = 0.0002) [257]

Liver cancer	*In serum (colorimetry)*: 119.8 U/mL compared to 153.0 U/mL (HBV positive controls) and 172.7 U/mL (healthy controls) (*p* < 0.001) [258] *In plasma (Oyanagui/Sun et al.)*: 13.5 ± 0.23 U/mL (stage I/II) and 10.7 ± 0.56 U/mL (stage III/IV) compared to 17.8 ± 0.14 in controls (*p* < 0.05) [231]	*In blood (Aebi)*: 16.094 ± 1.774 EU/g Hb (primary) and 13.599 ± 0.516 EU/g (metastatic) compared to 61.480 ± 0.210 in controls (*p* < 0.005) [259] *In plasma (Johansson and Borg)*: 8.26 ± 0.25 (stage I/II) and 5.07 ± 0.37 U/mL (stage III/IV) compared to 11.1 ± 0.32 in controls (*p* < 0.05) [231]	*In plasma (Paglia and Valentine)*: 125.60 ± 85.79 nmol/mL/min compared to 148.84 ± 92.77 in corresponding post-resection samples (ns) *In tissue (Paglia and Valentine)*: 41.43 ± 17.10 nmol/min/mg protein compared to 31.07 ± 10.95 nmol/min/mg in adjacent normal tissues (*p* < 0.05) [90] *In tissue (Paglia and Valentine)*: Se-GPx: 0.021 ± 0.009 *μ*mol/min/mg protein compared to 0.031 ± 0.015 *μ*mol/min/mg in adjacent normal tissues (*p* < 0.05) Non-Se-Gpx: 0.045 ± 0.021 *μ*mol/min/mg protein compared to 0.062 ± 0.02 *μ*mol/min/mg in adjacent normal tissues (*p* < 0.05) [143] *In plasma (Paglia and Valentine)*: 154 ± 18 U/L (stage I/II) and 82 ± 16 U/L (stage III/IV) compared to 266 ± 21 U/L in controls (*4*) [231]	*In plasma (Habig's et al.)*: 47.65 ± 22.51 nmol/mL/min compared to 38.85 ± 16.27 nmol/mL/min in corresponding post-resection samples (*p* < 0.05) *In tissue (Habig's et al.)*: 60.61 ± 75.51 nmol/min/mg protein compared to 70.94 ± 37.97 nmol/min/mg in adjacent normal tissues (ns) [90] *In tissue (Habig's et al.)*: 0.019 ± 0.012 *μ*mol/min/mg protein compared to 0.03 ± 0.013 *μ*mol/min/mg in adjacent normal tissues (*p* < 0.05) [143]

Ovarian cancer	*In erythrocytes (Marklund and Marklund)*: 454.67 ± 44.82 U/g Hb (stage II) and 316.86 ± 75.8 (stage IV) compared to 607.06 ± 154.08 in controls (*p* < 0.001) [236] *In erythrocytes (Misra and Fridovich)*: 672.2 ± 57.1 U/g Hb compared to 645.1 ± 40.9 U/g in controls (*p* < 0.05) [238]	*In serum (Goth)*: 28.2 ± 15.5 nmol/L/min compared to 36.1 ± 14.6 nmol/L/min in controls (*p* = 0.019) [237] *In erythrocytes (Beers and Sizer)*: 6.4 ± 1.3 U/g Hb compared to 7.2 ± 1.4 in controls (*p* < 0.01) [238]	*In serum (GPX3—ELISA)*: 22.4 ng/mL compared to 27.8 in controls (*p* = 0.01) [260] *In erythrocytes (Paglia and Valentine)*: 50.3 ± 1.2 U/g Hb compared to 48.7 ± 1.1 U/g in controls (*p* < 0.001) [238]	*In plasma (Habig et al.)*: 13.2 ± 0.6 *μ*mol/dL compared to 9.2 ± 0.9 *μ*mol/dL in controls (*p* < 0.001) [238]

Prostate cancer	*In whole blood (McCord and Fridovich)*: 24 U/mg protein compared to 22 U/mg in controls (*p* < .0.05) [Figure 1(d)] [171] *In erythrocytes (Misra and Fridovich)*: 1292.7 ± 534.6 U/g Hb compared to 1017.9 ± 253.5 in controls (ns) [67]	*In whole blood (Nelson and Kiesow)*: 1.3 U/mg protein compared to 1.5 U/mg in controls (*p* < 0.05) [Figure 1(c)] [171] *In erythrocytes (Beers and Sizer)*: 62.48 ± 13.79 10^4^ IU/g Hb compared to 58.62 ± 10.04 IU/g in controls (ns) [67]	*In blood (N/A)*: 540 ± 158 U/L compared to 675 ± 163 U/L in controls (*p* < 0.005) [240] *In erythrocytes (Paglia and Valentine)*: 8.1 ± 3.9 U/g Hb compared to 12.2 ± 6.3 U/g in controls (*p* < 0.001) [67]	*In serum (Habig et al.)*: 410 ± 174 U/mL.min compared to 307 ± 151 U/mL.min in controls (*p* < 0.005) [240]

Skin cancer	*In erythrocytes (Sun et al.)*: 1846.925 U/g Hb compared to 4547.013 U/g in controls (*p* < 0.0001) [241] *In cells (Roth and Gilbert)*: 0.52 ± 0.1 U/10^6^ cells compared to 0.41 ± 0.05 in normal melanocytes (*p* < 0.05) [79] *In erythrocytes (Marklund and Marklund)*: 7.376 ± 0.479 (T1/T2) and 9.037 ± 1.026 U/g Hb × 10^3^ (T3/T4) compared to 8.551 ± 0.499 in controls (ns) [75] *In plasma (Oyanagui/Sun et al.)*: 2.55 ± 0.43 U/mg protein compared to 4.38 ± 0.80 in controls (*p* < 0.001) *In tissue (MnSOD—IHC)*: 1.41 ± 0.65 IRS compared to 1.95 ± 0.53 in controls (*p* < 0.05) and 2.60 ± 0.46 in adjacent epidermis (*p* < 0.001) [107] *In serum (Sun et al.)*: 1300 U/mL compared to 1200 U/mL in controls (ns) [Figure 2] [242]	*In erythrocytes (Beers and Sizer)*: 39282.81 U/g Hb compared to 14051.35 U/g in controls (*p* < 0.0001) [241] *In cells (Beers and Sizer)*: 1.02 ± 0.2 U/10^6^ cells compared to 3.03 ± 0.2 in normal melanocytes (*p* < 0.001) [79] *In erythrocytes (Aebi)*: 10.23 ± 0.603 (T1/T2) and 10.74 ± 0.653 absorption/min/g Hb (T3/T4) compared to 10.7 ± 0.534 in controls (ns) [75] *In plasma (Johansson and Borg)*: 2.55 ± 0.43 U/mg protein compared to 4.38 ± 0.80 U/mg in controls (*p* < 0.001) *In tissue (IHC)*: 1.18 ± 0.74 IRS compared to 1.79 ± 0.63 in controls (*p* < 0.05) and 2.00 ± 0.48 in adjacent epidermis (*p* < 0.001) [107] *In serum (Goth)*: 22 kU/L compared to 17 kU/L in controls (*p* < 0.01) [Figure 4] [242]	*In plasma (Paglia and Valentine)*: 0.42 ± 0.13 U/mg protein compared to 0.77 ± 0.20 U/mg in controls (*p* < 0.001) *In tissue (IHC)*: 1.24 ± 0.51 IRS compared to 2.02 ± 0.37 in controls (*p* < 0.001) and 21.73 ± 0.41 in adjacent epidermis (*p* < 0.01) [107]	*In erythrocytes (Habig et al.)*: 1.815 ± 0.883 U/g Hb compared to 2.002 ± 0.529 U/g in controls (ns) [261]

Thyroid cancer	*In tissue (Marklund and Marklund)*: 63 ± 11 (follicular carcinoma) and 65 ± 10 U/g tissue (papillary carcinoma) compared to 58 ± 9 in controls (*p* < 0.05) [244] *In tissue (Sun et al.)*: 3.66 ± 0.09 U/mg protein compared to 3.49 ± 0.11 U/mg in adjacent tissues (*p* < 0.0001) [245] *In erythrocytes (Kakkar)*: 1.64 ± 0.16 U/mg Hb compared to 2.14 ± 0.24 U/mg in controls (*p* < 0.001) [247]	*In tissue (Beers and Sizer)*: 586 ± 35 (follicular carcinoma) and 559 ± 31 U/g tissue (papillary carcinoma) compared to 564 ± 29 in controls (*p* < 0.05 and ns) [244] *In erythrocytes (Sinha)*: 4.42 ± 0.38 U/mg Hb compared to 5.86 ± 0.58 U/mg in controls (*p* < 0.001) [247]	*In tissue (Rotruck et al.)*: 22.6 ± 5.5 (follicular carcinoma) and 20.4 ± 3.9 U/min/g tissue × 10^3^ (papillary carcinoma) compared to 18.6 ± 3.2 in controls (*p* < 0.05) [244] *In tissue (Paglia and Valentine)*: 0.61 ± 0.07 U/mg protein compared to 0.97 ± 0.02 U/mg in adjacent tissues (*p* = 0.001) [245] *In plasma (Rotruck et al.)*: 186.13 ± 8.44 U/L compared to 222.68 ± 28.8 U/L in controls (*p* < 0.001) *In erythrocytes (Rotruck et al.)*: 26.6 ± 2.48 U/g Hb compared to 34.06 ± 3.5 U/g in controls (*p* < 0.001) [247]	*In leukocytes (Habig et al.)*: 0.713 ± 0.065 U/mg protein compared to 0.497 ± 0.063 U/mg in controls (*p* < 0.001) [262]

**Table 3:** Nonenzymatic antioxidant levels in clinical samples of cancer patients.

Type of cancer	Nonenzymatic antioxidant levels			
	Glutathione	Vitamin A (retinol, *β*-carotene)	Vitamin C (ascorbic acid)	Vitamin E (*α*-tocopherol)
Bladder cancer	*In tissue (Ellman)*: 7.11 ± 3.3 *μ*g/mg protein compared to 14.45 ± 4.11 *μ*g/mg in controls (*p* < 0.001) [214] *In plasma (Ellman)*: 5.14 ± 2.02 *μ*M compared to 6.04 ± 2.05 *μ*M in controls (*p* = 0.047) [263]	*In plasma (HPLC)*: 1.41 *μ*g retinol/mL compared to 1.53 *μ*g/mL in controls (*p* < 0.001) [264]	*In serum (Roe and Keuther)*: 0.19 ± 0.08 mg/dL compared to 0.51 ± 0.09 mg/dL in controls (*p* < 0.001) [183]	*In serum (fluorometry)*: 0.71 ± 0.19 mg/dL compared to 1.43 ± 0.09 mg/dL in controls (*p* < 0.001) [183] *In plasma (HPLC)*: 23.93 *μ*g/mL compared to 27.48 *μ*g/mL in controls (*p* < 0.001) [264]

Breast cancer	*In tissue (Tietze)*: 18.89 ± 4.21 mg/100 g tissue compared to 9.49 ± 3.23 mg/100 g in adjacent normal tissues (*p* < 0.001) [219] *In plasma (Ellman)*:27.58 ± 1.75 mg/dL compared to 32.11 ± 2.29 mg/dL in controls (*p* < 0.05) [218]	*In plasma (HPLC)*: 248.5 ng *β*-carotene/mL compared to 226.0 ng/mL in controls (*p* = 0.02) 626.4 ng retinol/mL compared to 602.9 ng/mL in controls (ns) [265]	*In plasma (Roe and Kuether)*0.63 ± 0.2 mg/dL compared to 1.1 ± 0.1 mg/dL in controls (*p* < 0.05) [218] *In plasma (Okamura)*: 0.68 ± 0.45 mg/dL compared to 1.09 ± 0.50 mg/dL in controls (*p* < 0.05) [266]	*In plasma (Baker et al.)*0.72 ± 0.19 mg/dL compared to 1.12 ± 0.11 mg/dL in controls (*p* < 0.05) [218] *In plasma (Hashim and Schuttringer)*: 0.92 ± 0.68 mg/dL compared to 1.73 ± 0.78 mg/dL in controls (*p* < 0.05) [266] *In plasma (HPLC)*: 11111.6 ng/mL compared to 10762 ng/mL in controls (ns) [265]

Cervical cancer	*In plasma (Ellman)*: 37 mg/dL compared to 21 mg/dL in controls (*p* < 0.001) [Figure 2] [224] *In plasma (Ellman)*: 1.5 mg/dL compared to 2 mg/dL in controls (*p* < 0.0001) [Figure 4] *In erythrocytes (Ellman)*: 35 mg/dL compared to 48 mg/dL in controls (*p* < 0.0001) [Figure 6] [119] *In plasma (Hu)*: 269.66 ± 82.48 *μ*mol/mL compared to 316.18 ± 74.09 *μ*mol/mL in controls (*p* < 0.001) [225]	*In serum (HPLC)*: 2.04 *μ*mol retinol/L compared to 2.14 *μ*mol/L in controls (ns) 1.08 *μ*mol *β*-carotene/L compared to 1.34 *μ*mol/L in controls (*p* < 0.01) [267]	*In plasma (Roe and Kuether)*: 0.3 mg/dL compared to 1.1 mg/dL in controls (*p* < 0.001) [Figure 2] [224] *In plasma (Roe and Kuether)*: 0.7 mg/dL compared to 1.3 mg/dL in controls (*p* < 0.0001) [Figure 3] [119]	*In plasma (Baker et al.)*: 0.8 mg/dL compared to 2.3 mg/dL in controls (*p* < 0.001) [Figure 2] [224] *In plasma (Baker et al.)*: 1 mg/dL compared to 2.3 mg/dL in controls (*p* < 0.0001) [Figure 3] [119] *In serum (HPLC)*: 17.1 *μ*mol/L compared to 17.3 *μ*mol/L in controls (ns) [267]

Colorectal cancer	*In tissue (GSH-400 method)*: 174 ± 36 (stage I), 156 ± 39 (stage II), and 150 ± 48 nmol/g tissue (stage III) compared to 167 ± 15 in normal colon mucosa (*p* < 0.05) [83] *In erythrocytes (Beutler et al.)*: 7.05 ± 1.61 nmol/g Hb compared to 11.20 ± 1.1 nmol/g in controls (*p* < 0.05) [253]	*In serum (HPLC)*: 0.62 ± 0.1 *μ*g retinol/mL compared to 0.6 ± 0.2 *μ*g/mL in controls (ns) 0.32 ± 0.3 *μ*g*β*-carotene/mL compared to 0.33 ± 0.33 *μ*g/mL in controls (ns) [268] *In plasma (HPLC)*: 0.807 ± 0.752 *μ*M retinol compared to 1.237 ± 0.610 *μ*M in controls (*p* = 0.0021) [174]	*In tissue (HPLC)*: 458 ± 88 (stage I), 399 ± 90 (stage II) and 325 ± 92 nmol/g tissue (stage III) compared to 513 ± 64 in normal colon mucosa (*p* < 0.001) [83] *In plasma (HPLC)*: 29.457 ± 27.414 *μ*M compared to 49.76 ± 29.24 *μ*M in controls (*p* = 0.0006) [174]	*In serum (HPLC)*: 21.87 *μ*mol/L compared to 21.69 *μ*mol/L in controls (ns) [226] *In tissue (HPLC)*: 33.1 ± 9.1 (stage I), 29.1 ± 9.4, (stage II), and 13.3 ± 10.3 nmol/g tissue (stage III) compared to 37.5 ± 5.2 in normal colon mucosa (*p* < 0.001) [83] *In serum (HPLC)*: 18.4 ± 11.2 *μ*g/mL compared to 16.6 ± 7.6 in controls (ns) [268] *In plasma (HPLC)*: 18.87 ± 14.5 *μ*M compared to 24.69 ± 14.55 *μ*M in controls (*p* = 0.049) [174]

Lung cancer	*In tissue (Ellman)*: 24.1 ± 12.0 nmol/mg compared to 13.6 ± 6.5 in adjacent normal tissue (*p* = 0.0004) [257]	*In serum (HPLC)*: 1.362 *μ*M retinol compared to 2.496 *μ*M in controls (*p* < 0.001) 0.026 *μ*M *β*-carotene compared to 0.166 *μ*M in controls (*p* = 0.002) [269] *In serum (HPLC)*: 2.76 ± 0.32 *μ*mol/L compared to 1.60 ± 0.35 in smoker controls (*p* ≤ 0.001) [270]	*In serum (Roe and Kuether)*: 48.26 ± 6.81 *μ*mol/L compared to 16.65 ± 4.46 in smoker controls (*p* ≤ 0.001) [270]	*In serum (HPLC)*: 14.07 *μ*M compared to 24.458 *μ*M in controls (*p* < 0.001) [269] *In serum (HPLC)*: 15.67 ± 3.67 *μ*mol/L compared to 14.66 ± 3.88 in smoker controls (ns) [270]

Liver cancer	*In plasma (Tietze)*: 38.86 ± 26.15 *μ*mol/L compared to 48.66 ± 30.17 *μ*mol/L in corresponding post-resection samples (*p* < 0.05) *In tissue (Tietze)*: 42.76 ± 20.59 *μ*mol/g protein compared to 29.17 ± 14.92 *μ*mol/g in adjacent normal tissues (*p* < 0.05) [90] *In tissue (Sedlak and Lindsay)*: 4.62 ± 2.94 *μ*mol/mg protein compared to 5.52 ± 3.27 in adjacent normal tissues (*p* < 0.05) [143] *In plasma (Tietze)*: 8.3 ± 2.3 *μ*mol/L (stage I/II) and 5.5 ± 3.0 *μ*mol/L (stage III/IV) compared to 12.8 ± 1.5 in controls (*p* < 0.05) [231]	*In serum (HPLC)*: 32.5 *μ*g retinol/dL compared to 41.8 *μ*g/dL in controls (*p* < 0.001) 9.44 *μ*g *β*-carotene/dL compared to 11.57 *μ*g/dL in controls (*p* = 0.001) [271] *In plasma (HPLC)*: 0.74 ± 0.23 *μ*mol*β*-carotene/L (stage I/II) and 1.05 ± 0.33 *μ*mol/L (stage III/IV) compared to 0.56 ± 0.14 *μ*mol/L in controls (ns and *p* < 0.05) [231]	*In plasma (Zannoni et al.)*: 24.8 ± 2.1 *μ*mol/L (stage I/II) and 17.9 ± 5.3 *μ*mol/L (stage III/IV) compared to 31.7 ± 3.6 *μ*mol/L in controls (*p* < 0.05) [231]	*In plasma (HPLC)*: 22.8 ± 3.4 (stage I/II) and 24.1 ± 2.7 *μ*mol/L (stage III/IV) compared to 19.2 ± 2.8 in controls (*p* < 0.05) [231] *In serum (HPLC)*: 8.13 *μ*g/dL compared to 8.79 *μ*g/dL in controls (*p* = 0.02) [271]

Ovarian cancer	*In erythrocytes (Beutler et al.)*: 11.7 ± 2.9 mg/g Hb compared to 18.7 ± 2.7 mg/g in controls (*p* < 0.001) [238]	*In plasma (HPLC)*: 59.8 *μ*g retinol/dL compared to 68.6 *μ*g/dL in controls (*p* = 0.0183) 13.6 *μ*g *β*-carotene/dL compared to 21.5 *μ*g/dL in controls (*p* < 0.0001) [272]	*In erythrocytes (Roe and Keuther)*: 4.1 ± 1.2 mg/dL compared to 4.5 ± 1.3 mg/dL in controls (*p* < 0.001) [238]	*In serum (Baker et al.)*: 0.93 ± 0.18 mg % (stage II) and 0.72 ± 0.12 (stage IV) compared to 1.10 ± 0.15 in controls (*p* < 0.01 and *p* < 0.001) [236] *In erythrocytes (Baker et al.)*: 6.9 ± 1.4 *μ*mol/L compared to 7.3 ± 1.4 *μ*mol/L in controls (*p* < 0.01) [238] *In plasma (HPLC)*: 1.09 mg/dL compared to 1.34 mg/dL in controls (*p* = 0.0005) [272]

Prostate cancer	*In plasma (Ellman)*: 1.8 *μ*mol/mL compared to 1.45 *μ*mol/mL in controls (*p* < .0.05) [Figure 2(a)] *In erythrocytes (Ellman)*: 1.87 *μ*mol/mL compared to 1.37 *μ*mol/mL in controls (*p* < 0.05) [Figure 2(b)] [171] *In blood (Beutler et al.)*: 36.75 ± 3.9 mg % compared to 42.73 ± 3.3 mg % in controls (*p* < 0.001) [240]	*In plasma (HPLC)*: 580.3 ng retinol/mL compared to 565.6 ng/mL in controls (*p* = 0.02) [273]	*In serum (Roe and Keuther)*: 360 *μ*mol/mL compared to 520 *μ*mol/mL in controls (*p* < 0.05) [Figure 2(c)] [171]	*In serum (Hansen and Warwick)*: 12 *μ*mol/mL compared to 16 *μ*mol/mL in controls (*p* < 0.05) [Figure 2(d)] [171] *In plasma (HPLC)*: 10809 ng/mL compared to 11068 ng/mL in controls (ns) [273]

Skin cancer	*In erythrocytes (Tietze)*: 80.27 ± 6.836 (T1/T2) and 73.06 ± 5.227 *μ*M/g Hb (T3/T4) compared to 169.3 ± 23.02 *μ*M/g in controls (*p* = 0.0006 and *p* = 0.0005) [75] *In plasma (Tietze)*: 235.76 ± 42.75 *μ*mol/mg protein compared to 152.19 ± 44.88 *μ*mol/mg in controls (*p* < 0.001) [107] *In plasma (Ellman)*: 0.321 ± 0.123 mmol/L compared to 0.447 ± 0.094 mmol/L in controls (*p* < 0.0001) [261]	*In plasma (HPLC)*: 725.3 ng retinol/mL compared to 722.9 ng/mL in controls (ns) 153.2 ng *β*-carotene/mL compared to 168.2 ng/mL in controls (ns) [274] *In tissue (HPLC)*: 0.00939 ± 0.00219 nmol*β*-carotene/mg tissue compared to 0.00941 ± 0.00266 nmol/mg in adjacent tissue (ns) [175]	*In plasma (HPLC)*: 59.95 *μ*M (stage I), 58.85 *μ*M (stage II), 57.27 *μ*M (stage III), and 47.16 *μ*M (stage IV) compared to 64.86 *μ*M in controls (ns for stages I-III; *p* < 0.0001 for stage IV) [177] *In tissue (HPLC)*: 0.67473 ± 0.19749 nmol/mg tissue compared to 0.82778 ± 0.2214 in adjacent tissue (*p* = 0.022) [175]	*In cells (GC-MS)*: 5.83 ± 0.25 ng/10^6^ cells compared to 3.38 ± 0.5 in normal melanocytes (*p* < 0.005) [79] *In tissue (HPLC)*: 0.00977 ± 0.00214 nmol/mg tissue compared to 0.00947 ± 0.00264 nmol/mg in adjacent tissue (ns) [175] *In plasma (HPLC)*: 12.8 *μ*g/mL compared to 13.1 *μ*g/mL in controls (ns) [274]

Thyroid cancer	*In tissue (Beutler et al.)*: 2.8 ± 0.1 (follicular carcinoma) and 3.4 ± 0.4 *μ*M/g tissue (papillary carcinoma) compared to 2.9 ± 0.5 in controls (ns and *p* < 0.05) [244] *In serum (Ellman)*: 244.34 ± 27.0 *μ*mol/L compared to 377.87 ± 37.39 in controls (*p* < 0.05) [248] *In plasma (Beutler et al.)*: 38.2 ± 3.02 mg/dL compared to 48.7 ± 3.76 in controls (*p* < 0.001) *In erythrocytes (Beutler et al.)*: 39.3 ± 2.6 mg/dL compared to 52.6 ± 4.1 in controls (*p* < 0.001) [247]	N/A	*In plasma (Roe and Keuther)*: 0.64 ± 0.06 mg/dL compared to 1.56 ± 0.15 mg/dL in controls (*p* < 0.001) [247]	*In plasma (Desai)*: 0.85 ± 0.08 mg/dL compared to 1.41 ± 0.13 in controls (*p* < 0.001) *In erythrocytes (Desai)*: 1.58 ± 0.14 *μ*g/mg protein compared to 2.08 ± 0.19 in controls (*p* < 0.001) [247]

**Table 4:** Total antioxidant status in clinical samples of cancer patients.

Type of cancer	Total antioxidant status
Bladder cancer	*In serum (Koracevic method)*: 0.99 ± 0.06 mM compared to 1.45 ± 0.22 mM in controls (*p* < 0.001) [183] *In urine (DPPH)*: 1.15 mM vitamin C equivalent compared to 1.4 mM in controls (*p* < 0.001) [Figure 3(a)] [215] *In urine (ABTS)*:1.26 ± 0.63 mM/mM creatinine compared to 2.05 ± 0.46 mM/mM Cr. (*p* < 0.001) [263]
Breast cancer	*In serum (ABTS)*: 3.25 mmol/L compared to 2 mmol/L in controls (*p* < 0.001) [Figure 3] [217] *In plasma (ABTS)*: 1.35 mmol/L compared to 1.6 mmol/L in controls (*p* < 0.05) [Figure 1] [275]
Cervical cancer	*In serum (ABTS)*: 1.32 ± 0.029 mM compared to 1.62 ± 0.042 mM in controls (*p* < 0.001) [276]
Colorectal cancer	*In serum (ABTS)*: 1.60 mmol Trolox equivalent/L (stage I; *p* < 0.006), 1.44 mmol/L (stage II; *p* < 0.001), 1.53 mmol/L (stage III; *p* < 0.001), and 1.54 mmol/L (stage IV; *p* < 0.001) compared to 1.77 mmol/L in controls [277]
Lung cancer	*In serum (BAP)*: 2208 ± 314.8 *μ*mol/L in never-smokers and 2397 ± 323.7 *μ*mol/L in ever-smokers [44] *In serum (ABTS)*: 1.52 mmol Trolox equivalent/L compared to 1.78 mmol/L in controls (*p* < 0.001) [254]
Liver cancer	*In plasma (ABTS)*: 4421.72 ± 616.52 *μ*mol Trolox equivalent/L compared to 4593.42 ± 496.29 *μ*mol/L in corresponding post-resection samples (ns) *In tissue (ABTS)*: 256.84 ± 82.76 *μ*mol Trolox equivalent/g protein compared to 201.29 ± 58.38 *μ*mol/g in adjacent normal tissues (*p* < 0.05) [90] *In plasma (ABTS)*: 410 mg vitamin C equivalent/L compared to 460 mg/L in controls (*p* < 0.0001) [Figure 1(b)] [233]
Ovarian cancer	*In serum (ABTS)*: 1.33 ± 0.17 mmol Trolox equivalent/L compared to 1.58 ± 0.15 mmol/L in controls (*p* < 0.001) [278]
Prostate cancer	*In serum (ABTS)*: 2.56 ± 0.49 mmol Trolox equivalent/L [279]
Skin cancer	*In plasma (TRAP)*: 38.31 ± 4.209 *μ*M Trolox (T1/T2) and 35.72 ± 4.469 (T3/T4) compared to 30.38 ± 2.7 in controls (ns) [75]
Thyroid cancer	*In serum (ABTS)*: 1.37 ± 0.20 mmol Trolox equivalent/L compared to 1.67 ± 0.15 in controls (*p* < 0.0001) [192] *In serum (ABTS)*: 1.13 ± 0.03 mmol Trolox equivalent/L compared to 1.24 ± 0.02 mmol/L in controls (*p* = 0.011) [243] *In serum (FRAP)*: 284.5 ± 39.93 *μ*M compared to 486.00 ± 76.62 *μ*M in controls (*p* < 0.05) [248]
